# Evaluation of ambient mass spectrometry tools for assessing inherent postharvest pepper quality

**DOI:** 10.1038/s41438-021-00596-x

**Published:** 2021-07-01

**Authors:** Tyler J. Mason, Harmonie M. Bettenhausen, Jacqueline M. Chaparro, Mark E. Uchanski, Jessica E. Prenni

**Affiliations:** Department of Horticulture and Landscape Architecture, Colorado State University, Fort Collins, CO 80524 USA

**Keywords:** Metabolomics, Mass spectrometry

## Abstract

Horticulturists are interested in evaluating how cultivar, environment, or production system inputs can affect postharvest quality. Ambient mass spectrometry approaches enable analysis of minimally processed samples under ambient conditions and offer an attractive high-throughput alternative for assessing quality characteristics in plant products. Here, we evaluate direct analysis in real time (DART-MS) mass spectrometry and rapid evaporative ionization-mass spectrometry (REIMS) to assess quality characteristics in various pepper (*Capsicum annuum* L.) cultivars. DART-MS exhibited the ability to discriminate between pod colors and pungency based on chemical fingerprints, while REIMS could distinguish pepper market class (e.g., bell, lunchbox, and popper). Furthermore, DART-MS analysis resulted in the putative detection of important bioactive compounds in human diet such as vitamin C, *p*-coumaric acid, and capsaicin. The results of this study demonstrate the potential for these approaches as accessible and reliable tools for high throughput screening of pepper quality.

## Introduction

Inherent postharvest quality characteristics such as nutritional content and flavor are important to the perceived value of vegetable crops, but they are not often measured. Horticulturists are particularly interested in evaluating the impact of production system inputs and farming management practices on the quality of plant products such as flowers, fruits, and seeds. In addition, breeders are working to advance experimental lines that have exceptional flavor^1^ or high nutritional content^2^. Ultimately, these efforts are driven by the goal of increasing the production of high value vegetable crops, which could provide a competitive advantage for growers, especially those selling directly to the end-user such as restaurants or shoppers at a farmers market.

Evaluation of sensory qualities that impact consumer preference (e.g., appearance, texture, and flavor) is notoriously challenging. Many studies have begun to include on-farm sensory evaluations as an approach to better understand consumer expectations for vegetable quality attributes. While this approach can be successful, consumer focused sensory panels are expensive, in terms of both time and resources, and the information that is collected is relatively subjective^3,4^, limiting their widespread use. Thus, there is a need for novel approaches to assess vegetable quality that do not rely on human sensory evaluation.

Human perception of overall flavor is influenced by interactions between taste, aroma, mouthfeel, sight, and sound^5^. The chemical composition of non-volatile compounds contributes primarily to taste, whereas volatile compounds reflect aroma^6^. Analysis platforms that can collect qualitative and quantitative chemical data offer a way to objectively characterize vegetable quality attributes^7^ that are reflective of the human sensory experience. In addition, these techniques have the added advantage of being able to detect bioactive compounds that cannot be assessed through sensory approaches. For example, *p*-coumaric acid, an important phenolic compound with suggested beneficial bioactivity, has previously been detected in peppers by mass spectrometry^8^.

Mass spectrometric techniques such as gas chromatography mass spectrometry (GC-MS)^9^, coupled with solid phase micro extraction (SPME)^7^, and liquid chromatography mass spectrometry (LC-MS) have been utilized to characterize, screen, and differentiate between pepper species^10^. For example, SPME-GC-MS has been used to profile the volatile compounds contributing to aroma in peppers^7^. GC-MS has been utilized to characterize pepper accessions from the Embrapa Clima Temperado active germplasm, which identified a subset of accessions that were defined by higher fructose abundance^11^. LC-MS analysis of multiple pepper species (*C. annuum*, *C. chinense*, *C. frutescens*, and *C. baccatum)* was able to link chemical profiles with pungency^12^.

While these previous studies demonstrate that mass spectrometric techniques could detect compounds reflective of vegetable quality, they required extensive sample preparation, use of expensive instrumentation, long analysis times, and high technical expertise, representing practical barriers to adoption. For example, analyses of pepper samples by SPME-GC-MS typically involves some form of sample homogenization and extraction to enable sample uniformity^13^. In addition, once processed, sample analysis can take up to 30 min followed by extensive data processing and analysis, all of which requires a trained laboratory technician. These types of limitations as well as the high cost of adoption often put such approaches out of reach for horticulture studies. Thus, there is a need for alternative approaches that facilitate easy, rapid, and cost-effective analysis of vegetables for objective screening of quality attributes.

Ambient mass spectrometry platforms off an attractive alternative as they can operate under ambient conditions and require minimal sample preparation, thereby enabling a high-throughput method for quality analysis^14^. For example, ambient mass spectrometry platforms such as rapid evaporative ionization mass spectrometry (REIMS) and direct analysis in real time mass spectrometry (DART-MS) have been successfully used to evaluate, screen, and differentiate between a variety of sample types. REIMS has been used in the biomedical industry to screen for cancerous tissue in real time during operations^15^, to distinguish between fish species^16^, to assess porcine meat quality^17^, and to classify quality attributes such as grade and muscle tenderness in beef^18^. Similarly, DART-MS has been utilized to detect monoterpenes, sesquiterpenes, flavonoids, and organic compounds in the leaf and stem tissue between four *Eucalyptus* species^19^. A recent review article indicated DART-MS was also able to detect arecaidine, arecoline, and guvacoline (bioactive compounds) in plant tissues^20,21^. Novotná et al. (2012) analyzed extracted tomato and pepper samples from crops grown under organic and conventional management over two years using DART-MS coupled with time of flight (TOF). Interestingly, they observed that the chemical fingerprint generated by DART-MS was better able to predict growing season than production system^22^.

The overall goal of this study was to evaluate REIMS and DART-MS for their potential to perform rapid screening of peppers and classification based on quality. Our approach coupled the analytical acquisition of chemical profiles with chemometrics to generate predictive models for phenotypic quality parameters such as pod color, flavor, and bioactive compounds. The results presented here lay important groundwork for future incorporation of these tools into agricultural research workflows.

## Results and discussion

### DART-MS

**Fig. 1:**
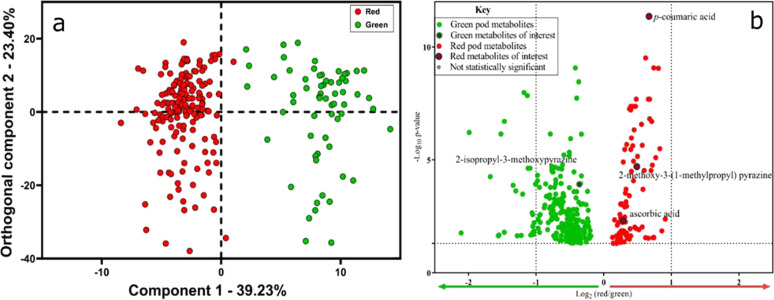
Vizualization of multivariate and univariate statistical analysis ofDART-MS data. (**a**) multivariate O2PLS-DA scores plot by color (red vs. green) (**b**) volcano plot from univariate analysis showing the statistically significant (Benjamini-Hochberg false-discovery rate adjustments and *α* = 0.05) differences between mass bins associated with red and green bell peppers

The multivariate O2PLS-DA modeling (red vs green) of the chemical profiles generated by DART-MS demonstrate that 39.2% of the variation in the data can be explained by pod color (Fig. 1a). The O2PLS-DA model exhibited an overall model fit of 0.89 (R^2^) and a cross-validated predictive accuracy of 75% (Q^2^). Overall, 92 mass bins were determined to be significantly different (Benjamini-Hochberg false-discovery rate adjustment and *α* *=* 0.05; Fig. 1b) and of these, 25 were putatively annotated based on comparison to compounds previous detection in peppers and DART-MS analysis of authentic standards when possible (Table 1, Figs. S1-S2).

**Table 1:** Putative compound annotations from DART-MS analysis of peppers

m/z bin	Putative identification
104.26	Malonic acid
120.26	Tetrose
121.26	p-aminobenzaldehyde
136.26	Limonene
160.76	Aminoadipic acid
164.26	*p*-coumaric acid^a^
166.26	2-methoxy-3-(1-methylpropyl) pyrazine
174.26	Dehydroascorbic acid
176.26	Ascorbic acid (vitamin C)^a^
216.26	Alpha hydroxylauric acid
219.26	Pantothenic acid
240.26	N-heptadecane
254.26	4-methylheptadecane
268.26	Nonadecane
269.26	Capsiamide
277.26	Dinorcapsaicin
282.26	Oleic acid
286.26	Luteolin
291.26	Norcapsaicin
305.26	Capsaicin^a^
313.26	N-cis-feruloyltyramine
313.76	Moupinamide
319.26	Homocapsaicin
321.26	Homodyhydrocapsaicin

^a^Supported by DART-MS analysis of authentic standards (Fig. S2). All other putative annotations based on evidence of previous detection in peppers

The mass bin putatively annotated as 2-isopropyl-3-methoxypyrazine was observed to be significantly (*p* = 0.002) enriched in green pepper pods, a result that agrees with previous studies using SPME-GC-MS^23–25^. Two of the mass bins detected in red pepper samples were putatively annotated as *p*-coumaric acid and ascorbic acid (vitamin C) (Fig. 1). Vitamin C has been previously reported to accumulate in mature red peppers and studies have also reported detection of *p*-coumaric acid in red peppers^26^. Two additional mass bins were putatively annotated as capsaicin and 2-methoxy-3-(1-methylpropyl) pyrazine, both compound that have been previously detected in peppers and contribute to pepper sensory and flavor quality^4,9^.

**Fig. 2:**
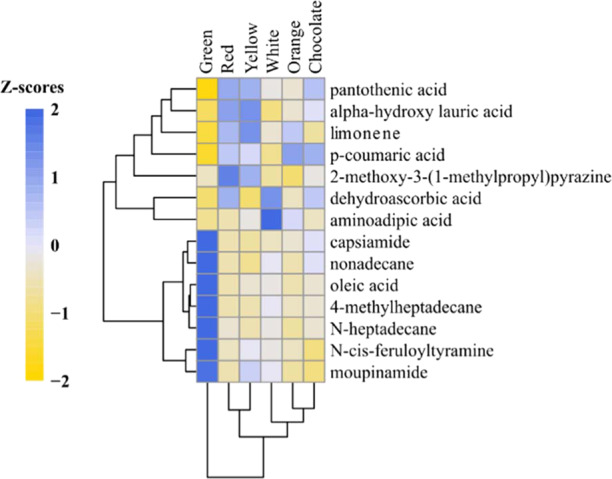
Heat map showing the differences in putatively annotated mass bins from DART-MS analysis of green, white, red, yellow, chocolate, and orange bell pepper phenotypes

Using only the putatively annotated mass bins, a comparison of the six different bell peppers analyzed by DART-MS indicates subgrouping (based on hirarchial clustering) of red and yellow and orange and chocolate (Fig. 2). Green followed by white bell peppers were the most distant among all phenotypes. Interestingly, chocolate colored bell peppers contained the highest abundance of the mass bin putatively annotated as p-coumaric acid. Yellow bell peppers contained the highest abundance of the mass bin putatively annotated as limone, a terpene compound that has a “citrus” aroma^27^. Red bell peppers contained the highest abundance of the mass bin putatively annotated as 2-methoxy-3-(1-methylpropyl) pyrazine, which is described to have a “musty, earthy, peppery” aroma^28^.

The trend of more N-heptadecane in green peppers has been reported previously in the literatue^26^. Our results indicate significantly higher abundance of mass bins putatively annotated as nonadecane and oleic acid in green peppers compared to red peppers, a result that diverges from what has been reported in the previous studies^29,30^. Nonadecane is a compound that serves as a maturity indictor. It has been reported to be absent in the volatile fraction of green bells, whereas it has the highest levels at the ripening stage and lower levels at maturity. Thus, our observation of higher abundance of nonadecane in green peppers (based on putative mass bin annotation) compared to mature red peppers, suggests that some of the green pods likely were developing a chemical fingerprint that was beginning to resemble ripening. Given that peppers were harvested for up to three weeks prior to analysis, it is possible that some of the green pods continued to mature postharvest. The observed trends for the mass bins putatively annotated as N-heptadecane and alpha-hydroxylauric acid, align with the literature^22^ for bell pepper pod color comparisons.

**Fig. 3:**
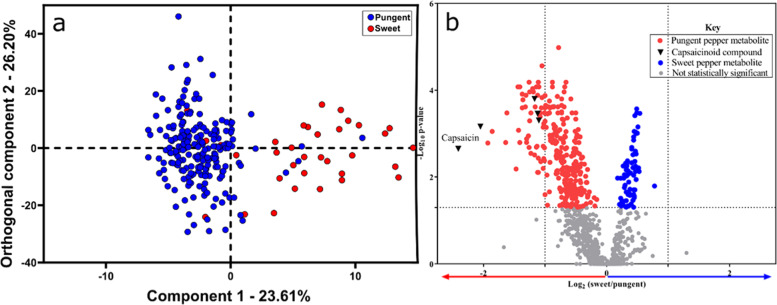
Vizualization of multivariate and univariate statistical analysis ofDART-MS data. (**a**) multivariate O2PLS-DA scores plot by flavor (sweet vs. pungent) (**b**) volcano plot from univariate analysis showing the statistically significant (Benjamini-Hochberg false-discovery rate adjustments and *α* = 0.05) differences between mass bins associated with sweet and pungent peppers

DART-MS was also able to detect qualitative differences between sweet and pungent peppers. The O2PLS-DA model exhibited an overall model fit of 0.79 (R^2^) and a cross validated predictive accuracy of 60% (Q^2^). Figure 3 illustrates that the distinction between sweet and pungent peppers (component 1) explained 23.6% of the variability in the data, and the orthogonal component was able to explain 26.2% of the variability. Using the Benjamini-Hochberg false-discovery rate adjustment, the mass bins putatively annotated as capsaicin (*p* = 0.002), homocapsaicin (*p* = 0.0003), homodihydrocapsaicin (*p* = 0.001), norcapsaicin (*p* = 0.004), and dinorcapsaicin (*p* = 0.0001) were significantly more abundant in the pungent peppers.

### REIMS

**Fig. 4:**
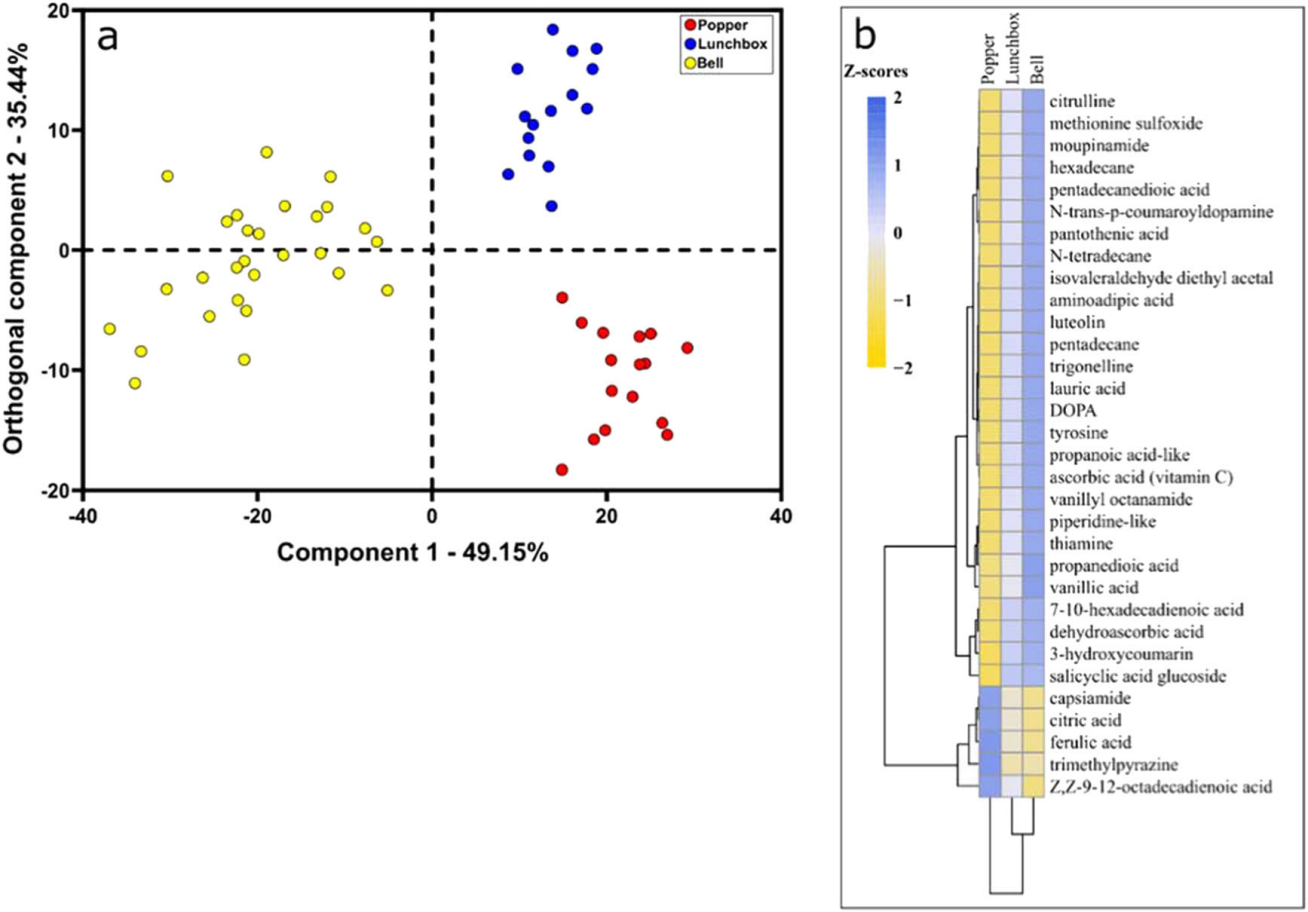
Vizualization of multivariate and univariate statistical analysis of REIMS data. (**a**) multivariate O2PLS-DA scores plot by market class (**b**) heat map showing the relative differences (z-scores) in chemical profiles of popper, lunchbox and bell peppers

The multivariate O2PLS-DA modeling of the chemical profiles generated by REIMS demonstrates that 49% of the variation in the data can be explained by market class (Fig. 4a). The O2PLS-DA model (market class) exhibited an overall model fit of 0.94 (R^2^) and a cross-validated predictive accuracy of 74% (Q^2^). Overall, 201 mass bins were determined to be statistically significant (Benjamini-Hochberg false-discovery rate adjustment and *α* = 0.05) and of these, 32 were putatively annotated based on previous detection in peppers (Table 2). A representative mass spectrum genereated by REIMS is presented in Fig. S3. Previous studies have demonstrated that chemical profiles generated by GC-MS grouped together according to pungency rather than species^12^.

**Table 2:** Putative compound annotations from peppers analyzed by REIMS

m/z bin	Putative identification
74.24	Propanoic acid
104.24	Propanedioic acid
122.24	Trimethylpyrazine
137.24	Trigonelline
160.24	Isovaleraldehyde diethyl acetal
161.24	Aminoadipic acid
162.24	3-hydroxycoumarin
165.24	Methionine sulfoxide
168.24	Vanillic acid
174.24	Dehydroascorbic acid
175.24	Citrulline
176.24	Ascorbic acid (vitamin C)
181.24	Tyrosine
192.74	Citric acid
194.74	Ferulic acid
197.24	DOPA
198.24	n-tetradecane
200.24	Lauric acid
212.24	Pentadecane
217.24	Pyrimidine like
219.24	Pantothenic acid
226.24	Hexadecane
252.24	7,10-hexadecadienoic acid like
265.24	Thiamine
269.74	Capsiamide
272.24	Pentadecanedioic acid
279.24	Vanillyl octanamide
280.74	(Z,Z)-9,12-octadecadienoic acid like
286.24	Luteolin
299.24	n-trans-p-coumaroyloctopamine
300.24	Salicylic acid glucoside
313.24	Moupinamide

Annotations are based on evidence from previous detection in peppers

Interestingly, the bell pepper market class contained the highest abundance of the mass bins putatively annotated as vitamin C and its precursor dehydroascorbic acid. Bell peppers also contained the highest abundance of the mass bin putatively annotated as luteolin, which is a flavonoid with antioxidant and anti-inflammatory activity^25^. The chemical fingerprint for the popper market class was the most different from the bell market class, while the lunchbox peppers were most similar to the bell pepper market class (Fig. 4). The highest abundance of the mass bins putatively annotated as capsiamide, citric acid, ferulic acid, and octadecadienoic acid was observed in the popper market class. Ferulic acid, belongs to a class known as coumaric acids which are important bioactive compounds with the ability to scavenge free radicals^26^. In addition, poppers also contained the highest relative abundances of the mass bin putatively annotated as trimethylpyrazine, which is known to have a “nutty” aroma and “musty” flavor^27^.

The two ambient mass spectrometry platforms evaluated in this study differed in their abilities to detect pepper quality characteristics. While these technologies do not enable highly accurate compound annotation, the putative annotations based on comparison with previous literature and known pepper compounds suggest that the chemical fingerprints reflect compounds relevant to sensory and nutritional quality. For example, DART-MS was able to putatively detect compounds known to contribute to aroma between red and green peppers (e.g., 2-methoxy-3-(1-methylpropyl) pyrazine) as well as compounds associated with fresh-eating quality characteristics (e.g., pungency) and bioactive compounds (e.g., *p*-coumaric acid). REIMS was able to putatively detect compounds of nutritional interest and quality related compound differences between red pepper phenotypes; the mass bin putatively annotated as luteolin, a bioactive compound with anti-inflammatory activity^30^ had the highest abundance in red bell peppers. In addition, REIMS also detected differences in the mass bin putatively annotated as vanillic acid, a compound known to be associated with a “smooth, vanilla” type aroma^24^. Taken together, the results of this study demonstrate that both platforms were able to detect volatile and non-volatile compounds important in the characterization of inherent quality attributes. Furthermore, the preliminary predictive models suggest the practical potential of these approaches for the development of screening assays (based on larger training sets) that could be implemented in agricultural studies to enable high throughput evaluation of pepper quality.

## Materials and methods

### Plant material for DART-MS

Pepper *(C. annuum*) seeds from 40 different cultivars (Table S1) were sown on 24 March 2019 into plastic plug trays (50 cell, Harris Seeds, Rochester, NY) containing a mixture of 40 L of soilless media (Sunshine mix #4, Sun Gro Horticulture, Agawam, MA), 10 L of worm castings, 250 ml of blood meal, and 250 ml of bone meal. The average daily temperature in the greenhouse was 25 °C and the relative humidity was 50%. The transplants were hardened by placing them outside under an insect-netting covered high tunnel with the east and west end walls removed for 7 days prior to transplanting. Pepper plants were grown between 5 June and 3 Oct. 2019 in a field at the Agricultural Research, Development and Education Center (ARDEC) South in Fort Collins, CO (lat. 40°36’N., long. 104°59’W.) elevation 1,524 m. The pepper cultivars were planted 0.5 m apart in black plastic mulched beds, which were spaced 1.8 m apart. Macronutrient needs (57 kg/acre nitrogen) were met with monthly applications of Drammatic “One” (4-4-0.5) fertilizer (Dramm Corporation, Manitowoc, WI) through the drip irrigation system. Black plastic drip tape emitting water at a rate of 500 L/h/100 m with emitters spaced 20 cm apart was used to irrigate the crop. Using an irrigation controller, we provided 15–30 min of irrigation once or twice daily. The crop was scouted for pests in the field on a weekly basis. Fully mature pepper pods were harvested on 9 and 23 Sept. as well as on 3 Oct. 2019. The pods were stored in 7 °C walk-in cooler until quality characteristics could be evaluated via DART-MS. Fresh pods were taken to the lab where they were stored in a 7 °C refrigerator until they could be rinsed with deionized water and dried with disposable paper towels. Pepper pungency was determined by the cultivar description in grower seed catalogs or by speaking with the pepper breeder.

### DART-MS analysis

The experiment was run as a randomized complete block design with eight replicates spanning 8 days. One representative pepper pod served as the replicate. After every 8th sample, a quality control (QC) sample was analyzed. The QCs (6/day) consisted of a sub-sample from the same pod belonging to “Ace”. A disposable utility razor blade was used to cut a 7 cm long by 2 cm wide slice of pepper longitudinally down the center of the pod just prior to analysis. From this piece, 2 mm thick cross-sectional cuts were made to expose the exocarp, mesocarp, and endocarp tissue. The sliced pepper samples were laid sideways on the tablet carrier adapter for the sample introduction rail system (IonSense, Inc., Saugus, MA).

The DART-MS analysis was conducted using the DART-Standardized Voltage and Pressure (DART-SVP) model ion source (IonSense, Inc., Saugus, MA). It was coupled to a single quadrupole mass spectrometer (ACQUITY QDa; Waters Corporation, Manchester, UK) via a Vapor interface (IonSense, Inc., Saugus, MA). The DART-SVP was equipped with a motorized linear rail where the tablet carrier was mounted. The helium flow rate for the ion source was set to ~3 L/min heated to 350 °C. The cone voltage was set to 20 V. Spectra were acquired in negative ionization mode over the mass range of 50–500 m/z. The speed of the motorized linear rail system holding the tablet carrier adapter was set to 1.0 mm/sec. The standby temperature was held at 245 °C. The samples were arranged on the 10 Tablet™ module (metal rail) such that three pepper sub-samples from the same pod were placed in every other tablet location. This allowed the signal from each sample to return to the baseline before the next acquisition could be started.

Authentic analytical standards for *p*-coumaric acid, capsaicin, and L-ascorbic acid were analyzed using the same instrument conditions with the exception of the sample introduction (≥98% purity) (Sigma-Aldrich, St. Louis, MO). A 10 mg/mL solution was made for L-ascorbic acid was using HPLC grade water. Solutions (10 mg/mL) were made for *p*-coumaric acid, trans-*p*-coumaric acid, and capsaicin using 100% ethanol. Twelve replicates of each sample were run through the DART using the DIP-IT™ method. This involved dipping individual 10 µL glass capillary rods into each solution and placing the rods into the DIP IT™ module holder, which allowed the analytical standards to pass directly in front of the ionizing source.

Putative annotations were assigned by cross-referencing the m/z mass bins (0.5 m/z bin size) observed after pre-processing (described below) of the DART-MS spectra against all compounds that have been previously detected in red bell peppers as defined in the food database (FoodB, version 1) and comparison to standards when possible^31^.

### DART-MS data processing and statistical analysis

Preprocessing was conducted using a beta version of WRC Abstract Model Builder (Waters Corporation, Manchester, UK). Scans corresponding to the pepper sample spectra were selected and the signal was summed to generate one spectrum per sample. Data was normalized to the total ion current for each sample. Peak binning was conducted at an interval of 0.5 m/z (Table S2).

The effects of pod color, market class, cultivar, and pungency on the chemical profile were evaluated using analysis of variance (ANOVA) with the *aov* function in the R statistical environment (version 3.6.2)^32,33^. Using the *p.adjust* function in R, we used a Benjamini-Hochberg false discovery rate adjustment to identify mass bins of statistical significance at the *α* = 0.05 level. Orthogonal partial least squares discriminant analysis (O2PLS-DA) was performed using SIMCA (version 15) on unit variance scaled and LOG_10_ transformed data. The volcano plots were constructed using GraphPad (version 8.1.0). The x-axis indicates the differential abundance between red and green pepper pods; it was calculated using log_2_ red abundance divided by log_2_ green abundance. The y-axis indicates statistical significance using –log_10_ FDR adjusted *p* values (*α* = 0.05) for 333 metabolites. Sphere colors, red and green, indicate metabolite class. The vertical dashed lines are the threshold for red/green [log_2_(red/green) < -1.0 or > 1.0]. The volcano plot for the sweet and pungent pepper metabolite comparison was constructed using a similar approach where sweet was divided by pungent for 368 metabolites. The heat map, which is based on z-scores for each pepper pod color and putatively annotated mass bins, was generated using the *pheatmap* function in R. Z-scores were calculated by comparing the average relative abundance value for a mass bin to the population mean and population standard deviation for that mass bin.

### Plant material for REIMS

Approximately 24 pepper samples were purchased for each of the two market classes, bell and popper (Table S3) from Whole Foods Market (Fort Collins, CO) on 12 Feb. 2018. On the same day, 5 bags of ~24 tri-color (red, yellow, orange) sweet mini lunchbox pepper phenotypes were purchased from King Soopers (Kroger supermarket, Fort Collins, CO).

### REIMS analysis

Analysis was performed as a randomized complete block design with 16 replicates. The individual pepper phenotypes were cut into thirds. The exocarp of the pod was placed flat on the conduction pad so that the mesocarp was exposed. The “iKnife” was held perpendicular to the mesocarp tissue so the vacuum component could draw in the smoke generated by the ionizing source.

The chemical fingerprint was detected using the protocol described^18^. Briefly, the samples were analyzed using a Synapt G2 Si Q-ToF, fitted with a REIMS ionization source attached to a monopolar electrosurgical hand piece called an “iKnife” (Waters Corporation, Manchester, UK). It was powered with an Erbotom ICC 300 electrosurgical generator (Erbe Elektromedizin GmbH, Turbingen, Germany) using the “liquid coagulation” mode at a power of 40 W. A solution of 2 ng/mL leucine-enkephalin at a continual flow rate of 200 µL/min was directed to the REIMS source during sampling. The heater bias was set to 80 V and the cone voltage was set to 20 V. At least 3 “burns” were collected from each sample within a 3 cm × 3 cm square from the center of the pod. Each burn lasted ~3 s. Spectra were collected from 50 to 1200 m/z using positive ionization mode.

### REIMS data processing and statistical analysis

Pre-processing was performed using a beta version of Waters Abstract Model Builder. At least three peaks corresponding to three “burns” were selected and the signal was summed to generate one spectrum per sample. Data was normalized to the total ion current for each sample. Peak binning was conducted at an interval of 0.5 m/z over the m/z range from 50 to 550 m/z (Table S4).

The effects of market class on the chemical profile were evaluated using analysis of variance (ANOVA) with the *aov* function in the R statistical environment^32^. Using the *p.adjust* function in R, we used a Benjamini-Hochberg false discovery rate adjustment to identify mass bins of statistical significance at the *α* = 0.05 level. O2PLS-DA was performed using SIMCA (version 15) on unit variance scaled and LOG_10_ transformed data. The heat map illustrating differences in chemical profiles for each market class was generated using z-scores and the *pheatmap* function in R. Mass bins were putatively annotated using a similar method as to what was described above for DART-MS.

## Supplementary information

Supplemental Figures

Table S1

Table S3

Table S2

Table S4

## Data Availability

Pre-processed data matrices used for statistical analysis and to generate predictive models are available in supplementary information (Tables S1 and S2). Raw data for both DART-MS and REIMS experiments are available upon request.
