# Supplementary material for: Evaluation of ambient mass spectrometry tools for assessing inherent postharvest pepper quality
Source: Hortic Res. 2021 Jul 1;8:160. doi: 10.1038/s41438-021-00596-x (PMC8245583; doi:10.1038/s41438-021-00596-x)
Supplement: Supplementary file 1 — Supplemental Figures [file 41438_2021_596_MOESM1_ESM.pptx]

## Slide 1
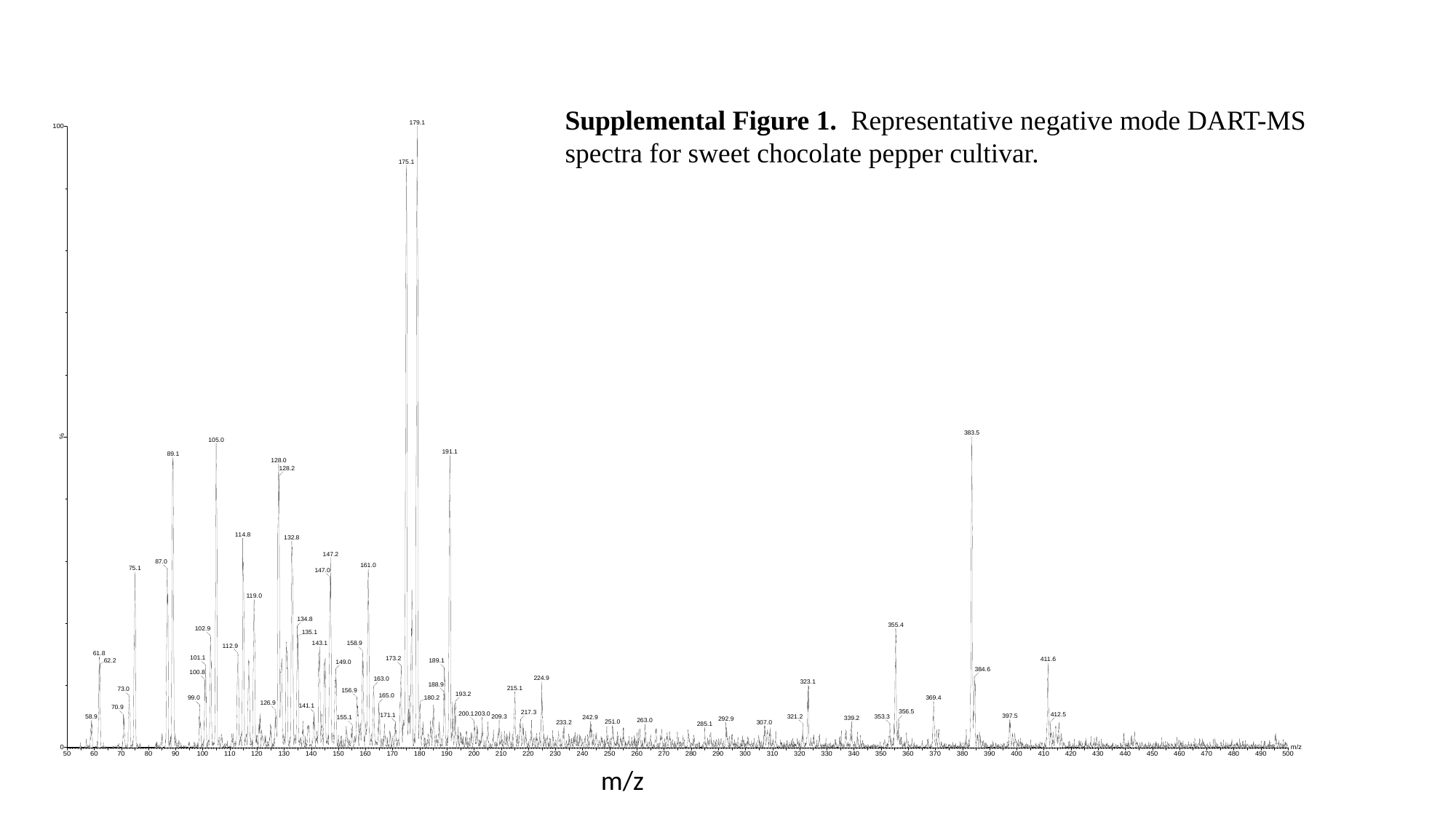

Supplemental Figure 1. Representative negative mode DART-MS spectra for sweet chocolate pepper cultivar.
m/z

## Slide 2
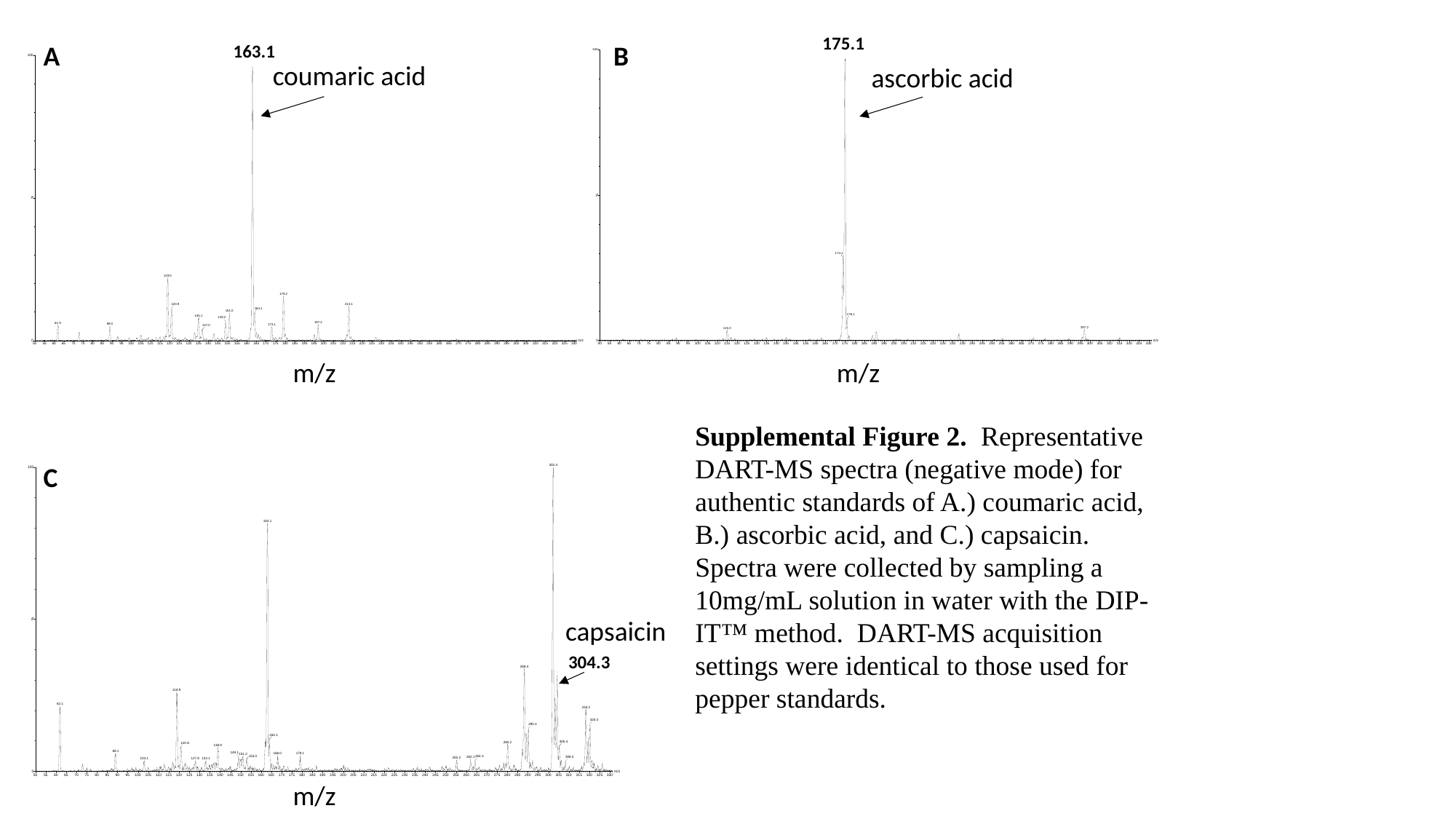

175.1
A
B
163.1
coumaric acid
ascorbic acid
m/z
m/z
Supplemental Figure 2. Representative DART-MS spectra (negative mode) for authentic standards of A.) coumaric acid, B.) ascorbic acid, and C.) capsaicin. Spectra were collected by sampling a 10mg/mL solution in water with the DIP-IT™ method. DART-MS acquisition settings were identical to those used for pepper standards.
C
capsaicin
304.3
m/z

## Slide 3
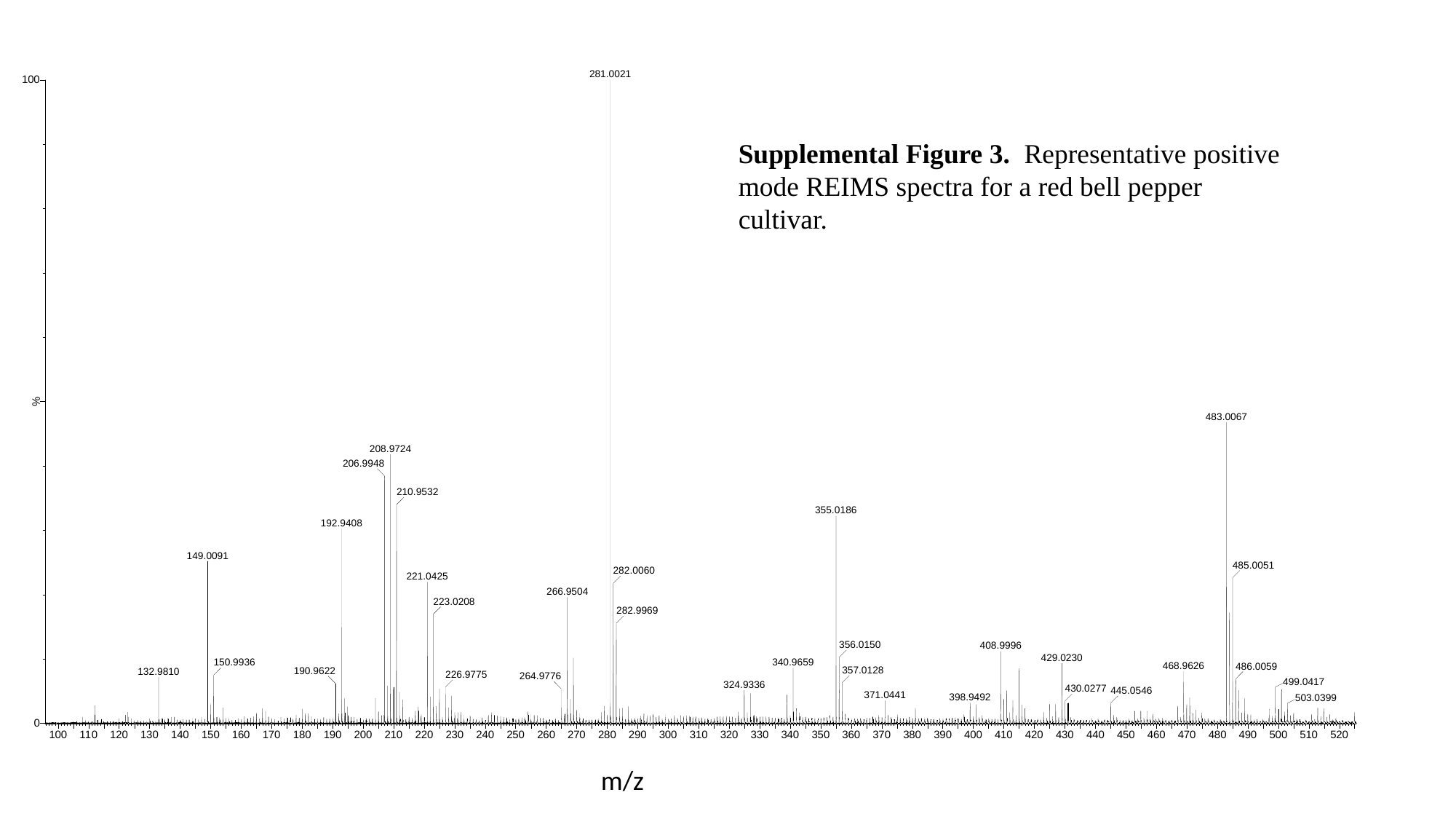

Supplemental Figure 3. Representative positive mode REIMS spectra for a red bell pepper cultivar.
m/z
