# Supplementary material for: Evaluation of ambient mass spectrometry tools for assessing inherent postharvest pepper quality
Source: Hortic Res. 2021 Jul 1;8:160. doi: 10.1038/s41438-021-00596-x (PMC8245583; doi:10.1038/s41438-021-00596-x)
Supplement: Supplementary file 2 — Table S1 [file 41438_2021_596_MOESM2_ESM.docx]

**Table S1.** The pod types, pod colors, and pungency for cultivars evaluated by DART-MS.

| **Cultivar** | **Pod type** | **Pod color** | **Pungency** |
| --- | --- | --- | --- |
| ‘Abay’ | Bell | Yellow | Sweet |
| ‘Ace’ | Bell | Red | Sweet |
| ‘Aristotle’ | Bell | Green | Sweet |
| ‘Aristotle’ | Bell | Red | Sweet |
| ‘Bianca’ | Bell | White | Sweet |
| ‘Early Red Sweet’ | Bell | Red | Sweet |
| ‘Flavorburst’ | Bell | Yellow | Sweet |
| ‘Jupiter’ | Bell | Red | Sweet |
| ‘Karisma’ | Bell | Red | Sweet |
| ‘King of the North’ | Bell | Red | Sweet |
| ‘Orange Marmalade’ | Bell | Orange | Sweet |
| ‘Peacework’ | Bell | Red | Sweet |
| ‘Procraft’ | Bell | Red | Sweet |
| ‘Sunrise’ | Bell | Yellow | Sweet |
| ‘Whitney’ | Bell | White | Sweet |
| ‘Wisconsin Lakes’ | Bell | Red | Sweet |
| ‘Yankee Bell’ | Bell | Red | Sweet |
| ‘Sweet Chocolate’ | Bell | Chocolate | Sweet |
| ‘Anaheim College 64’ | Italian-style | Green | Pungent |
| ‘Anaheim TMR #9457' | Italian-style | Green | Pungent |
| ‘Bridge to Paris’ | Italian-style | Red | Sweet |
| ‘Cañoncita Field 7 Landrace’ | Italian-style | Red | Pungent |
| ‘Carmen’ | Italian-style | Red | Sweet |
| ‘Colorado State University 321’ | Italian-style | Green | Pungent |
| ‘Colorado State University 384’ | Italian-style | Green | Pungent |
| ‘Colorado State University 390’ | Italian-style | Green | Pungent |
| ‘Colorado State University Mosco’ | Italian-style | Red | Pungent |
| ‘Early Perfect Italian’ | Italian-style | Red | Sweet |
| ‘Escamillo’ | Italian-style | Yellow | Sweet |
| ‘Highlander’ | Italian-style | Red | Pungent |
| ‘Joe E. Parker’ | Italian-style | Green | Pungent |
| ‘Karma’ | Italian-style | Red | Sweet |
| ‘Liebesapfel’ | Italian-style | Green | Pungent |
| ‘Melrose’ | Italian-style | Red | Sweet |
| ‘Stocky Red Roaster’ | Italian-style | Red | Sweet |
| ‘Sweet Delilah’ | Italian-style | Red | Sweet |
| ‘Yellow Bardo’ | Italian-style | Yellow | Sweet |
| ‘Paradicsom Alaku Sarga Szentes’ | Pimento | Yellow | Sweet |
| ‘Red Ruffled Pimento’ | Pimento | Red | Sweet |
| ‘Colorado State University Pueblo Popper’ | Popper | Red | Pungent |
