# Supplementary material for: Evaluation of ambient mass spectrometry tools for assessing inherent postharvest pepper quality
Source: Hortic Res. 2021 Jul 1;8:160. doi: 10.1038/s41438-021-00596-x (PMC8245583; doi:10.1038/s41438-021-00596-x)
Supplement: Supplementary file 3 — Table S3 [file 41438_2021_596_MOESM3_ESM.docx]

**Table S3.** The market classes and colors for pepper phenotypes evaluated using

REIMS.

| **Cultivar/Phenotype** | **Market Class** | **Color** |
| --- | --- | --- |
| 'Aloha' | Bell | Red with yellow stripes |
| Red sweet mini | Lunchbox | Red |
| Yellow sweet mini | Lunchbox | Yellow |
| Red popper | Popper | Red |
